# Increase consumers’ willingness to pay a premium for organic food in restaurants: Explore the role of comparative advertising

**DOI:** 10.3389/fpsyg.2022.982311

**Published:** 2022-08-03

**Authors:** Weiping Yu, Xiaoyun Han, Fasheng Cui

**Affiliations:** Business School, Sichuan University, Chengdu, PR, China

**Keywords:** comparative advertising, organic food, benefit appeal, willingness to pay a premium, persuasiveness, organic skepticism

## Abstract

Offering organic food is a new trend in the hospitality industry seeking sustainable competitiveness. Premiums and information barriers impede continued growth in organic consumption. This study aims to explore the role of comparative advertising (CA) in organic food communication. Three empirical studies were used to verify the effect of CA vs. non-comparative advertising (NCA) on consumers’ willingness to pay a premium (WTPP) for organic food, examining how benefit appeals (health vs. environmental) and consumers’ organic skepticism affects CA. The results indicate that matching CA and health appeals increase consumers’ WTPP, while environmental appeals have no significant differences between the CA and NCA groups (Study 1). Information persuasiveness mediates the interaction between CA and benefit appeal on WTPP (Study 2). CA increases WTPP among consumers with high organic skepticism, while the interaction between CA and health appeal is only effective for low skepticism consumers (Study 3). The findings unravel and explain the mechanics of how CA works in organic products, which can help restaurants, retailers and tourist destinations advertise organic food to increase consumers’ WTPP.

## Introduction

Current food production and consumption methods are averse to ecologically sustainable development. Globally, non-organic food production irresponsible for nearly one-third of all greenhouse gas emissions (Clark et al., 2020), while organic agriculture has the most negligible impact on environmental degradation (Vasile et al., 2015). Unlike traditional agriculture, the positive effects of organic agriculture on human health, environmental friendliness, and biodiversity have been confirmed (Mondelaers et al., 2009; Tandon et al., 2020), increasing its demand significantly (Talwar et al., 2021; Yu et al., 2021). A growing number of managers and academics are aware that offering organic food is a new trend in the hospitality industry seeking sustainable competitiveness (Cozzio et al., 2020; Zhang et al., 2021). Food consumption is a relatively easy and influential way to make a sustainable shift in tourism and hospitality (Jang et al., 2011; Yin et al., 2021).

Despite the advantages of organic food production, consumer appreciation, and consumer purchase propensity in surveys (Rana and Paul, 2017; Wang et al., 2021), its market share is less than 5% in most countries (Hansmann et al., 2020), and only 0.8% in China (China National Commercial Information Center, 2019; FiBL, and IFOAM, 2020). A significant gap exists between their willingness to buy and actual consumption (Carrington et al., 2014; Massey et al., 2018) because consumers neither realize nor believe in the claimed benefits of organic products (Van Doorn and Verhoef, 2015; Liu et al., 2021) or their high prices (Katt and Meixner, 2020). The gap is even more pronounced in the hospitality (Juvan and Dolnicar, 2014; Konuk, 2019) and when it comes to paying for organic food in restaurants (Jeong and Jang, 2019). Li et al. (2016) also found that nearly half of consumers were unwilling to pay more than 5% extra for sustainable products. Actually, the high production cost of organic food also implies that providers need to be paid a premium to avoid losses (Fanasch and Frick, 2020). Consumers’ willingness to pay a premium (WTPP) can better represent consumers’ real thoughts and action support for organic food. Increasing consumers’ WTPP has become an essential task for organic food producers, restaurants, and retailers (Jeong and Jang, 2019; Jäger and Weber, 2020).

Green advertising strategies play an irreplaceable role in promoting consumers’ WTPP for organic food (Aschemann-Witzel and Zielke, 2017; Wang et al., 2021). For example, after providing information about the impact of organic apple production on health and the environment, consumers were willing to pay more (Rousseau and Vranken, 2013). However, since it is difficult for consumers to verify the organic food benefits emphasized in green advertisements, people are increasingly skeptical of claims (Leonidou et al., 2014; Wei et al., 2022). An interesting phenomenon is that some organic food appeals will mention “other brands” products or similar traditional products (Harker, 2004), and compare product functional attributes or environmental attributes to express selfish or altruistic appeals (Schuhwerk and Lefkoff-Hagius, 1995; Kareklas et al., 2014). Previous research has found that informative appeals play an effective role in influencing guests’ choice of organic food in buffet (Cozzio et al., 2020). Organic food claims often activate consumer trust through comparisons (Schuhwerk and Lefkoff-Hagius, 1995; You et al., 2020).

Comparative advertising (CA) as a persuasion strategy is often used to compare one’s products or services with competitors in the same industry and highlight the “comparative advantage” of their products or services, thus influencing consumers’ preference (del Barrio-García et al., 2020). Studies have shown that CA leads to more persuasions and behavioral responses, but this is related to the product’s attribute (Bambauer-Sachse and Heinzle, 2018a). CA with verifiable attributes has been discussed extensively, such as product intrinsic and extrinsic attributes (Bambauer-Sachse and Heinzle, 2018b), or search and experience attributes (Jain et al., 2000). However, CA with non-verifiable attributes has not been clearly proven effective. For example, is it better to show that organic food is more nutritious than regular food? The underlying psychological mechanisms of how CA of credit attributes acts on consumers are not well understood.

Although consumers are aware of organic food’s health and environmental benefits, the premium price factor makes it difficult to make purchase decisions (Abraben et al., 2017; Jeong and Jang, 2019). Previous studies have mostly focused on factors affecting consumers’ willingness to buy (Kareklas et al., 2014; Tandon et al., 2020; Liu et al., 2021), only a few have focused on consumers’ WTPP. CA emphasizes comparative advantages (Abraben et al., 2017; Bambauer-Sachse and Heinzle, 2018a) that satisfy consumers’ expectations of higher quality organic products to make them willing to pay a premium (Yucel-Aybat and Kramer, 2018). Therefore, this study explores the effectiveness of CA in restaurant organic food claims and its impact on consumer WTPP.

Studies on the impact of advertising content and appeal methods on green purchase intention, suggest that environmental and health appeal are the two most common ways to advertise organic food (Tucker et al., 2012; Septianto et al., 2019). Environmental appeal emphasizes that organic food does not pollute soil and water resources and increases biodiversity (Jäger and Weber, 2020), while health appeal emphasizes that organic food is more natural, healthy, and nutritious (Zwier, 2009; Hidalgo-Baz et al., 2017). Although previous studies have explored these two appeals, the impact of their related attributes on consumers’ WTPP is inconclusive (Abraben et al., 2017; Haws et al., 2017; Septianto et al., 2019). The present study combines them with comparative claims to provide further verification.

## Theoretical background and hypotheses

### Comparative advertising

Comparative advertising is an advertisement in which a business operator compares a product/service directly or indirectly with one or more competitors (del Barrio-García et al., 2020). The commercial purpose of CA is to obtain a “comparative advantage” through comparative claims (Barry, 1993). CA can be divided into direct (specifically named competing brands) and indirect comparison advertising (“brand X,” “leading brand,” or “other alternative product”; Byun and Jang, 2018). Study on CA has grown rapidly in Asian countries in recent years (Kalro et al., 2017). Since CA is mainly indirect in China due to legal constraints, relevant studies are rare. The Advertising Law stipulates that advertisements shall not depreciate the goods or services of other producers or operators and that comparative advertisements must be based on factual evidence and not use any unfair or unscientific comparison methods^1^. Therefore, this study considers legitimate CA in the Chinese market as the research object.

Comparative advertising is not always more effective than non-comparative advertising (NCA), which usually induces more positive cognitive responses (Pechmann and Stewart, 1990) but less favorable emotional responses (Jeon and Beatty, 2002; Nagar, 2015). Based on the product type, specific attributes are classified intrinsically (e.g., taste or sweetness), and extrinsically (e.g., brand name or price). CA involving the extrinsic attribute of goods and services is more effective because it produces higher activation than intrinsic attribute comparisons (Bambauer-Sachse and Heinzle, 2018a). Scholars have verified the effectiveness of CA product attributes (i.e., experience and search). For experience attributes, NCA may be more effective than CA. However, for search attributes, the two advertisements may produce similar efficacy levels (Jain et al., 2000). When the typical attributes of a brand with high consumer commitment are the basis for comparison, NCA generates more favorable brand attitudes than CA. For atypical attributes, the two advertisement modes did not produce different brand attitudes [regardless of brand promises (Pillai and Goldsmith, 2008)].

Regarding content, consumers prefer comparative advertisements containing factual rather than evaluative or subjective information (Iyer, 1988), and the brand novelty of comparative ads leads to their effectiveness (Nye et al., 2008). Studies found that strong credibility of information sources is more favorable for CA than NCA (Kavanoor et al., 1997). In addition, an advertisement’s vague claims are more likely to be objectionable than a concrete comparison (Snyder, 1989; Bambauer-Sachse and Heinzle, 2018a).

In advertising comparisons, the competitor’s name is not directly mentioned in an indirect comparison, but in direct comparison, the market position of the competing brand and the market position and market share of the sponsoring brand, leading brand, and “multi-brand” comparison will affect the consumer’s response (Iyer, 1988; Kalro et al., 2017). Additionally, consumers’ characteristics will also affect the effectiveness of CA, such as gender (Chang, 2007), self-construal (Polyorat et al., 2005), information-processing style (Thompson and Hamilton, 2006), predisposition to show reactance (Bambauer-Sachse and Heinzle, 2018a), and culture (Choi and Miracle, 2004).

In sum, CA is mainly applied to products and services, and there is a lack of other category segmentation, while organic products suffer from obstacles in communication because of trust attributes (Nuttavuthisit and Thøgersen, 2017). Therefore, it is worth exploring whether advertising applies to organic products. In addition, the dependent variables of CA have mainly focused on advertising attitudes, brand attitudes, and purchase intention. This study examines whether CA can support organic food price premiums.

### Willingness to pay a premium

Willingness to pay a premium refers to a consumer’s willingness to pay more for a particular product than competing products (Chaudhuri and Holbrook, 2002). In this study, willingness to pay was associated with organic food. Usually, this represents the largest difference between consumers’ perceived value of organic and non-organic food (Katt and Meixner, 2020). Organic production is characterized by higher labor costs and lower yields than conventional production (Berghoef and Dodds, 2013). It provides a return only when consumers are willing to pay a premium to compensate for the high production costs (Fanasch and Frick, 2020). Meanwhile, a premium purchase is generally regarded as one of the strongest results of brand loyalty (Gounaris and Stathakopoulos, 2004). It is the value embodiment of customers’ perception of brand and quality. Therefore, we define WTPP as a consumer’s willingness to pay more for organic food of a particular brand than similar non-organic food.

Consumers’ WTPP for organic food is often restricted by distrust or inability to perceive the superiority of product quality (Nuttavuthisit and Thøgersen, 2017; Ladwein and Romero, 2021). Accordingly, organic food restaurants, or retailers use external signals to help consumers make purchase decisions (Cozzio et al., 2020; Fanasch and Frick, 2020). Particularly, to reduce information asymmetry, they can inform consumers that organic food is better than ordinary food. The driving force of price premiums is consumers’ desire for a certain product or service quality. For example, Lea and Worsley (2005) argued that organic food contains higher vitamin and mineral levels than ordinary food. More importantly, consumers associate healthier food with higher prices (Haws et al., 2017). CA, which emphasizes a product’s comparative advantage, is associated with more favorable attitudes and greater willingness to pay (Yucel-Aybat and Kramer, 2018). Therefore, the current study infers that CA plays a role in the WTPP for organic food.

### Interaction between comparative advertising and advertising benefit appeal

Increasingly, consumers express positive views about organic food, believing it is healthier and better for the environment than conventional food (Aschemann-Witzel and Zielke, 2017). While, many consumers won’t actually make a purchase (Carrington et al., 2014). The benefits of organic food are often not well communicated to consumers (Van Doorn and Verhoef, 2015), causing difficulty deciding on paying a premium. Marketers often use advertising that highlights self-benefits (such as nutrition and health) and other benefits (such as environmental protection and biodiversity) to promote sustainable products (Hidalgo-Baz et al., 2017; Septianto et al., 2019). Kareklas et al. (2014) demonstrated that advertising with both egoistic and altruistic appeals is as effective as altruistic (environmental) advertising, but both are more effective than egoistic (health) advertising. Conversely, Yadav (2016) found that while altruistic appeals promoted sustainable consumption, health claims were more important than environmental benefits in predicting young consumers’ organic food purchase intentions. Multiple studies show that health and environmental protection are the main motivations for consumers to buy organic food (Massey et al., 2018; White et al., 2019). Therefore, as a key “supplier-to-consumer” communication medium, it is important to highlight the health and environmental appeals of organic food (White and Peloza, 2009; Jäger and Weber, 2020). More practical information should be presented to enhance advertising effectivity (Byun and Jang, 2018).

Comparative advertising allows people to better understand the advantages of products by comparing them with competitive products, further making people more willing to pay a higher price (Yucel-Aybat and Kramer, 2018). The difference in organic advertising is that it usually compares to the ordinary, conventional, or non-organic food. This indirect comparison does not involve competing brands (Miniard et al., 2006). The advertisement’s content emphasizes two credit attributes specific to organic food (health and eco-friendliness; Nuttavuthisit and Thøgersen, 2017). Previous studies found that independent self-construal consumers are more likely to accept CA and generate purchase intention than interdependent self-construal consumers (Polyorat et al., 2005). The health claims of organic food are more related to the vital interests of consumers (Septianto et al., 2019), and the use of CA can better promote consumers’ awareness and WTPP (Haws et al., 2017). Regarding the environmental benefits of organic production, the impact on purchase intention is mixed (Chen and Chang, 2013). Some scholars believe that altruistic appeals effectively promote prosocial behavior when they activate public concern about self-image (White and Peloza, 2009). Meanwhile, environmental claims can stimulate consumers’ attitudes and purchase intention toward organic products (Kareklas et al., 2014). However, Visser et al. (2015) claimed that green advertising that focuses only on the environment would have a negative impact on purchase intention since environmental appeals (e.g., protection of the ecological environment, animal welfare, and biodiversity) are further removed from consumers’ lives (Thomas et al., 2021). Comparison methods may not inspire the persuasiveness of advertisements, and may even make consumers speculate on the motivations of businesses (Leonidou and Skarmeas, 2017). Therefore, the environmental appeal of CA is not necessarily more effective.

**H1:** CA claims and advertising benefit appeals have an interactive effect on consumers’ WTPP for organic food.

**H1a:** For health appeal, CA may have a stronger impact on consumers’ WTPP for organic food than NCA.

**H1b:** For environmental appeal, there is no difference in the impact of CA and NCA on the WTPP for organic food.

### Mediating role of information persuasiveness

Information persuasiveness refers to the ability of advertisements to persuade consumers to purchase and consume behaviors or to agree with certain viewpoints (Micu and Chowdhury, 2010). This means consumers trust the information and make relevant judgments and decisions based on it (Appelman and Sundar, 2016). The persuasiveness of the information to a consumer is determined by both advertising information and the consumers (Cesario et al., 2004). CA is considered more informative and stimulating because it provides stronger objective facts and persuasive evidence by comparing sponsored and competing brands according to specific attributes or market positions (Iyer, 1988; Barry, 1993; Nye et al., 2008).

For organic products, Information persuasiveness and credibility play a more important role because organic food is a credence product (Lee and Hwang, 2016). This means consumers cannot verify the promised benefits of the product before or after purchase (Lee and Hwang, 2016). Therefore, consumers need to believe that publicity in green advertising has become particularly important, as lack of trust in the benefits of sustainable products is the main barrier to paying a premium (Nuttavuthisit and Thøgersen, 2017; Britwum et al., 2021). The external information of food will affect consumers’ behavior, which will activate a cognitive persuasion mechanism to activate consumers’ willingness to pay (Teuber et al., 2016; Xiang et al., 2019; Wang et al., 2021). Information persuasiveness can help us better understand the information that can effectively impress consumers (Byun and Jang, 2018) and is the key to increasing sustainable consumption behavior (Jäger and Weber, 2020). CA and advertising appeals with different benefits may affect an advertisement’s persuasiveness. Thus, this study infers that the influence of these two variables on the WTPP is mediated by Information persuasiveness and proposes the following hypothesis:

**H2:** Information persuasiveness plays a mediating role in the combined effect of CA claims and advertising benefit appeals on the WTPP for organic food.

### Organic skepticism: Moderating mediating effect

Skepticism about advertising is a common tendency to disbelieve advertising claims and represents a basic market belief. This belief varies from person to person and is associated with general persuasiveness (Obermiller and Spangenberg, 1998). Based on the Persuasion Knowledge Model (PKM, Friestad and Wright, 1994), consumers can gain insight into marketing motivation and persuasion strategies *via* advertising. When persuasion knowledge is activated, consumers increase their suspicion of advertising and their perception of corporate manipulation (Jain et al., 2000). This suspicion diminishes when an advertisement’s persuasive strategy is credible (Byun and Jang, 2018). With the accelerated development of green marketing, the phenomenon of greenwashing occasionally occurs (Newman et al., 2014), leading to an increasing number of consumers doubting the environmental performance and benefits of sustainable products (Goh and Balaji, 2016). The current study introduces organic skepticism, a tendency for consumers to doubt the utility of organic products (Obermiller and Spangenberg, 1998; Leonidou and Skarmeas, 2017), that affects the persuasion of advertising claims (do Paço and Reis, 2012), encourages consumers to seek more product information, causes negative word-of-mouth (Leonidou and Skarmeas, 2017), reduces willingness to buy organic products, and reduces WTPP for products (Van Doorn and Verhoef, 2015).

However, consumers are less skeptical when an advertisement provides sufficient information (do Paço and Reis, 2012). Outstanding advertising claims can effectively reduce consumer uncertainty regarding product information (Han et al., 2015). Luo et al. (2020) found that green advertising on social media can negatively impact green purchase intention through the mediating effect of perceived information utility. Factors such as trust in the social system (Klopčič et al., 2020), health imagery (Chrysochou and Grunert, 2014), certification information (Janssen and Hamm, 2012), and visual aids (Hooker and Teratanavat, 2008) can influence consumers’ evaluation of products and stimulate purchases. Investigating how consumer skepticism affects responses to organic advertising claims can provide reliable and effective measures to reduce skepticism (Jäger and Weber, 2020). In addition, consumer skepticism about green messages is critical for understanding the effectiveness of organic advertising (Leonidou and Skarmeas, 2017). Consumer skepticism will curb green consumption (Goh and Balaji, 2016; Leonidou and Skarmeas, 2017), especially in the CA context, and is more likely to cause resistance. Thus, it is necessary to understand better how organic skepticism influences persuasion and willingness to pay premiums for organic food.

**H3:** Organic skepticism plays a moderating role in the combined effect of CA claims and advertising benefit appeals on Information persuasiveness.

**H4:** Organic skepticism moderates the mediating effect of persuasiveness on the WTPP through the interaction of CA claims and advertising benefit appeals.

**FIGURE 1:**
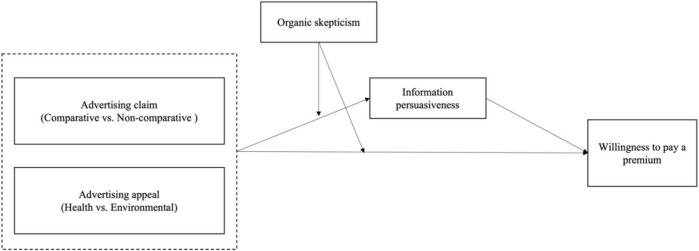
Theoretical model.

The theoretical model of this study is shown in Figure 1.

## Study 1: Effect of comparative advertising claims and benefit appeals effect on consumers’ willingness to pay a premium for organic food

Study 1 explored the interaction between CA claims and benefit type in green advertising on consumers’ WTPP for organic food. Consistent with **H1**, this study expected that CA would be more effective regarding health appeals, with no difference between comparative and NCA regarding environmental appeals.

### Participation and design

Chinese consumers most often choose organic food categories: vegetables, fruits, and dairy products. The total organic crop yield amounted to 13.356 million tons (Zhang et al., 2020). Therefore, tomato, apples, and milk were selected as the stimulating materials in this study. The study employed a 2 (type of claim: comparative, non-comparative) × 2 (type of benefit: health vs. environment) between-subjects design. Participants were 148 college students from a large university in Southwest China (39.2% males, 60.8% females). One of the questions requires the respondent to choose the third option to complete the screening. If respondents choose other options, they will not be included in the sample. A total of 126 valid samples were collected after 22 invalid samples failed to undergo attention screening.

### Procedure

A laboratory experiment was conducted to simulate the dining scene in a restaurant, and participants were given the choice of organic tomatoes at their meal and randomly presented with one of four ads for organic tomatoes. Regarding manipulating advertising benefit appeals, this study referred to real advertisements and previous studies (Newman et al., 2014). The organic advertisements were composed of images and text, and the difference between the four advertisements was only in their appeal content. Since CA involving competitors is controversial in China, this study used indirect comparisons (without reference to specific competitors). For example, the CA for health appeal was “Organic tomatoes are more nutritious and healthier than regular tomatoes. They do not use pesticides and are rich in vitamins and essential trace elements.” The environmental appeal emphasized the advantages over ordinary tomatoes regarding environmental protection.

After reading the stimulus information, participants were asked about their WTPP for the organic tomatoes advertised (Chaudhuri and Ligas, 2009). As for manipulation checks, the participants judged the degree to which the advertisement’s information related to health/environmental benefits (health/environment: 1 = none, 7 = very much); advertising used a comparative approach (1 = strongly disagree, 7 = strongly agree; Yang et al., 2015). Finally, the participants completed their personal information.

### Results

#### Manipulation checks

A one-way analysis of variance (ANOVA) was used to analyze the data. This study’s benefit appeal (health vs. environmental) design was effective: the health advertisements contained more information related to the health interests of consumers than environmental interests [*M*_*Health*_ = 4.52, *M*_*Environmental*_ = 3.56, *F* (1, 124) = 15.146, *p* < 0.001]. There was more information related to environmental protection in environmental advertisements than in health advertisements [*M*_*Health*_ = 2.42, *M*_*Environmental*_ = 4.77, *F* (1, 124) = 92.156, *p* < 0.001]. The comparative group scored significantly higher than the NCA group on whether the ads emphasized comparative advantage [*M*_*Comparative*_ = 4.84, *M*_*Non–comparative*_ = 4.06, *F* (1, 124) = 7.872, *p* < 0.005], indicating that advertisement manipulation was successful.

#### Willingness to pay a premium

**FIGURE 2:**
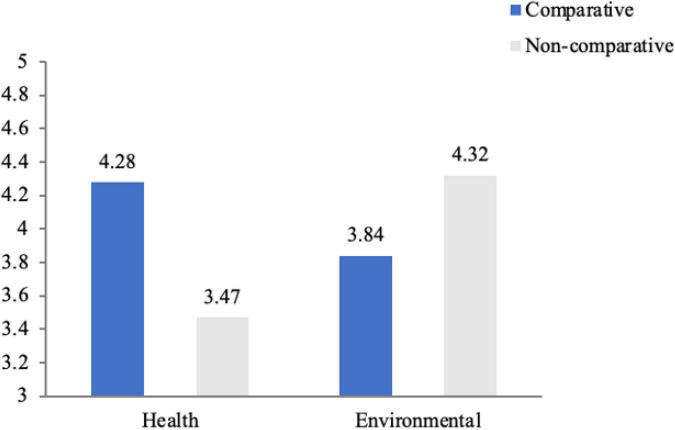
Interaction effect between CA claims and benefit appeals on willingness to pay a premium.

This study used WTPP as the dependent variable to verify the impact of the matching of CA and benefit appeal on the participants’ WTPP. The results indicated that the main effect of benefit appeal [*F* (1, 125) = 0.476, *p* = 0.491 > 0.10] and of CA [*F* (1, 125) = 0.662, *p* = 0.418 > 0.10] were not significant. However, the interaction between CA and benefit appeal had a significant impact on participants’ WTPP [*F* (1, 122) = 6.857, *p* = 0.01 < 0.05]. Therefore, **H1** is accepted. Furthermore, simple analysis revealed that compared with NCA, consumers were more willing to pay a premium for CA involving health benefits [*M*_*Comparative–Health*_ = 4.28, *M*_*Non–comparative–Health*_ = 3.47, *F* (1,122) = 5.474, *p* = 0.021 < 0.05], thus **H1a** is accepted. Compared with CA, consumers’ WTPP for NCA appealing to environmental benefits was relatively higher, [*M*_*Comparative–Environmental*_ = 4.32, *M*_*Non–comparative–Environmental*_ = 3.84, *F* (1,122) = 1.881, *p* > 0.1], but not significant (Figure 2); thus, **H1b** is accepted.

### Discussion

Study 1 verifies that the interaction between benefit appeal and CA significantly impacts the WTPP for organic food. The matching of CA and health appeal can prompt consumers to form a more active WTPP, and environmental appeals have a certain effect on premium payment regardless of the different types of CA. The subsequent experiments further explore the intermediary mechanisms and boundary conditions.

## Study 2: Mediating role of information persuasiveness

Study 2 chose organic apples as the stimulus material to further verify the interaction between advertising and advertising benefit appeals on the WTPP for organic food and the mediating role of information persuasiveness.

### Participation and design

Study 2 employed a 2 (type of claim: comparative, non-comparative) × 2 (type of benefit: health vs. environmental) between-subjects design. Therefore, to manipulate the type of benefits, participants were asked to read product information about organic apples’ health or environmental benefits. The information was expressed comparatively (e.g., “Organic apples are more nutritious and healthier than ordinary apples”) or non-comparatively (e.g., “Organic apples are nutritious and healthy”) to manipulate advertising claims. The stimulus material used the virtual organic apple brand “Xi Hong” and was presented *via* four advertisements that promoted organic apples using different expressions. The advertisements’ layout was consistent, including the product name, specific product attributes, and product image. Participants were selected from an online consumer panel *via* a Sojump survey^2^, the largest consumer database for online empirical research in China. The geographical structure of the sample is diverse, with consumers from cities such as Chengdu, Hangzhou, and Guangzhou participating. Fourteen subjects failed the attention screening procedure (the attention test was the same as study 1), and 177 respondents were recruited (48% male, 52% female, *M*_*age*_ = 28.31, SD = 8.72).

### Procedure

The survey simulated the scenarios of consumers purchasing apples in an organic grocerant shopping. Grocerant is a new concept that blends retail and restaurants (Kim et al., 2019). Participants were randomly assigned to one of four scenarios. After reading the advertisements, they responded to the manipulation test, information persuasiveness, perceived quality, WTPP, brand familiarity, product familiarity, and provided their basic information.

### Measurements

Information persuasiveness includes three items: “the information of this advertisement is credible; this information is convincing; the information of this advertisement affects my future choice of apple” (α = 0.882; Byun and Jang, 2018). To measure perceived quality (Jäger and Weber, 2020), participants were asked to answer a question: “I perceive the quality in this advertisement as?” (1 = low quality, 7 = high quality). WTPP, as referred from Chaudhuri and Ligas’s (2009), included two items: “I would be willing to pay a higher price for organic apples than ordinary apples; and I prefer to buy this organic apple, even if the prices of other apples are lower” (α = 0.873). Each item was scored on a 7-point Likert scale. The participants then completed their personal information (sex, age, education, and monthly income).

### Results

#### Manipulation checks

Through a one-way ANOVA, the healthy appeals contained more information related to consumers’ health benefits than environmental appeals [*M*_*Health*_ = 5.28, *M*_*Environmental*_ = 3.60, *F* (1,175) = 52.403, *p* < 0.001], and there was more information related to environmental protection in environmental appeals than in healthy appeals [*M*_*Health*_ = 2.42, *M*_*Environmental*_ = 4.6, *F* (1,175) = 85.416, *p* < 0.001]. The design of health vs. environmental benefit appeals was effective. There was no significant difference in brand familiarity [*M*_*Comparative*_ = 2.38, *M*_*Non–comparative*_ = 2.63, *F* (*1*, 175) = 1.173, *p* = 0.28] and product familiarity [*M*_*Comparative*_ = 3.21, *M*_*Non–comparative*_ = 3.32, *F* (*1*, 175) = 85.416, *p* = 0.703] between the comparative and non-comparative advertisement groups, indicating that participants were not affected by familiarity.

#### Willingness to pay a premium

A two-way ANOVA was conducted to verify the impact of the interaction between CA and benefit appeal on participants’ WTPP. The results demonstrate that the main effect of benefit appeal [*F* (1,176) = 0.162, *p* = 0.688 > 0.10] was not significant, but that of CA [*M*_*Comparative*_ = 3.978, *M*_*Non–comparative*_ = 3.597, *F* (1,176) = 3.153, *p* = 0.078 < 0.10] was significant within the 90% confidence interval. The interaction effect between benefit type and CA was significant [*F* (*1*, 124) = 6.857, *p* = 0.009 < 0.01]. Further simple analysis found that compared with NCA, consumers were more willing to pay a premium for the health benefits defined in CA [*M*_*Comparative–Health*_ = 4.22, *M*_*Non–comparative–Health*_ = 3.27, *F* (1,173) = 9.894, *p* = 0.002 < 0.01]; thus, supporting **H1a**. Environmental appeal had a certain effect on premium payment regardless of the CA type [*M*_*Non–comparative–Environmental*_ = 3.92, *M*_*Comparative–Environmental*_ = 3.74, *F* (1,173) = 0.348, *p* > 0.1]; thus, supporting **H1b**.

#### Mediating role of information persuasiveness

**FIGURE 3:**
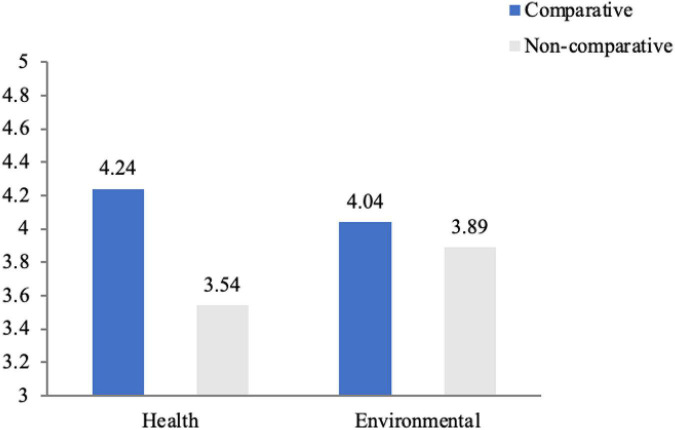
Interaction effect between CA claims and benefit appeals on information persuasiveness.

Information persuasiveness was used as a dependent variable for the preliminary analysis. As illustrated in Figure 3, the results manifested a significant interaction between CA and benefit appeal [*F* (*1*, 173) = 4.634, *p* = 0.033 < 0.05]. Regarding health benefits, consumers were more persuaded by advertisements using comparative claims [*M*_*Comparative–Health*_ = 4.24, *M*_*Non–comparative–Health*_ = 3.54, *F* (1, 173) = 6.28, *p* = 0.013 < 0.05]. Regarding environmental benefits, consumers had a higher evaluation of the persuasiveness of NCA, but the difference was not significant [*M*_*Non–comparative–Environmental*_ = 4.04, *M*_*Comparative–Environmental*_ = 3.89, *F* (*1*, 173) = 0.284, *p* > 0.1].

This study adopted the bootstrap method. Additionally, the PROCESS program in SPSS was used to examine the mediating role of persuasiveness in the interaction effect between CA (non-comparative = 0, comparative = 1) and benefit type (health appeal = 0, environmental appeal = 1) on WTPP.

The non-parametric percentile method of deviation correction was selected for sampling, the model was selected as 8, the sample size was set to 5,000, the confidence interval was set to 95%, and brand familiarity and product familiarity were included as covariates. The results showed that the moderated mediating effect confidence interval did not contain 0 (β = –0.7340, SE = 0.2854, LLCI = –1.3435, and ULCI = –0.2189), indicating a moderated mediating effect. After the mediation was controlled, the interaction effect between CA and benefit type had no effect on the dependent variable, and the interval contained 0 (β = –0.6575, SE = 0.1936, LLCI = 0.3052, and ULCI = 1.0707). The above results indicated that Information persuasiveness plays a mediating role; thus, **H2** is supported. Study 2 also supports **H1**, **H1a**, and **H1b**.

## Study 3: Moderating mediating role of organic skepticism

### Participation and design

Study 3 introduced the variable of organic skepticism to explore the boundary conditions of the theoretical model. It adopted an inter-group design of 2 (type of claim: comparative, non-comparative) × 2 (type of benefit: health vs. environment) × 2 (organic skepticism: low vs. high) to test the moderating mediating role of organic skepticism. The experimental stimulus material was pure organic milk, and participants were asked to read its product information. The virtual brand was “HEMU.” Similar to study 2, this experiment was conducted through an online survey. After screening (answer time less than 3 min or fail attention screening), 219 questionnaires were collected from participants (52% male, *M*_*age*_ = 30.62, SD = 7.86).

### Procedure

First, participants were tested on their level of organic skepticism. Then, they will see the following prompts: “Imagine you plan to drink pure milk and see an advertisement of organic milk.” Participants were randomly assigned to read the online advertisements in four scenarios, then responded regarding manipulation tests, advertisement persuasiveness, perceived quality, and WTPP. Willingness to pay was explored by asking: “How much more than ordinary pure milk are you willing to pay for HEMU organic pure milk (%)?” The participants then answered questions on organic advertising, brand familiarity, product familiarity and provided their basic information.

### Measurements

The measurements for Information persuasiveness (α = 0.902) and WTPP (α = 0.886) were the same as study 2. The measurement for organic skepticism was from Mohr et al. (1998) and Royne et al. (2012) and included four (modified) measurement items (α = 0.863): “The accuracy of organic food advertising is questionable; I do not trust organic food advertising; most organic food advertising is exaggerated; most organic food advertisements online are intended to mislead rather than to inform consumers.”

### Results

#### Manipulation checks

A one-way ANOVA found that the health vs. environmental design was effective. Healthy appeal advertising contained more green information related to consumers’ health benefits than environmental advertising [*M*_*Health*_ = 4.84, *M*_*Environmental*_ = 3.82, *F* (1, 217) = 22.006, *p* < 0.001]. There was a significant difference between the comparative and NCA groups in judging HEMU pure organic milk as better than plain pure milk, showing that CA is an effective design.

#### Willingness to pay a premium

To verify the impact of the matching of CA and benefit appeal on the participants’ WTPP, this study used willingness to pay as the dependent variable to perform an ANOVA. From the analysis of the interaction effect between benefit appeal and CA claims [*F* (1, 215) = 5.104, *p* = 0.025 < 0.05], the results were significant; thus, **H1** is supported again. Further simple analysis found that compared with NCA, consumers were more willing to pay a premium for health benefits defined in CA [*M*_*Comparative–Health*_ = 4.27, *M*_*Non–comparative–Health*_ = 3.25, *F* (1,215) = 13.242, *p* < 0.001]; thus, **H1a** is accepted. Compared with comparative advertisements, consumers had a higher WTPP for environmental benefits appealing to non-comparative advertisements, but the difference was not significant [*M*_*Non–comparative–Environmental*_ = 4.02, *M*_*Comparative–Environmental*_ = 3.90, *F* (1, 215) = 0.676, *p* > 0.1]; thus, **H1b** is supported.

#### Moderating effect of organic skepticism

With the mean value as the dividing point, organic skepticism was divided into high and low groups by adding or subdividing the standard deviation. A three-way ANOVA, with Information persuasiveness as the dependent variable, showed that the interactive effects of CA, advertising benefit appeal, and organic skepticism were significant [*F* (1,218) = 4.198, *p* = 0.042 < 0.05], indicating that the three factors had a common effect on consumers’ perceived Information persuasiveness; thus, **H3** is supported.

**FIGURE 4:**
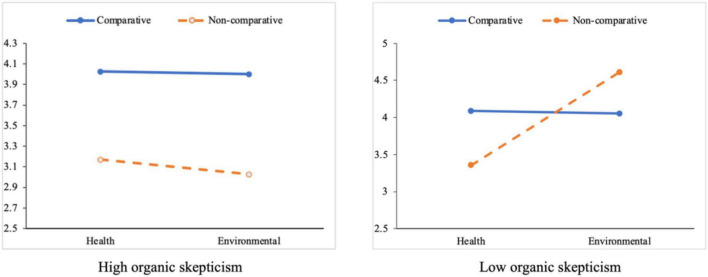
Information persuasiveness by CA claims, benefit appeal, and organic skepticism conditions.

For the high organic skepticism group, the interactive effect of CA and benefit type was not significant [*F* (1, 100) = 0.063, *p* = 0.802 > 0.01]. However, CA effectively convinced consumers with high organic skepticism (Figure 4). For the low organic skepticism group, the interaction between CA and benefit types was significant [*F* (1, 111) = 6.968, *p* = 0.009 < 0.01], indicating this interaction on Information persuasiveness was only effective among low organic skepticism consumers.

#### Moderating the mediating effect of organic skepticism

The bootstrap method was used to test how organic skepticism moderated the mediating effect of persuasiveness on the WTPP through the interaction of CA claims and advertising benefit appeals. The non-parametric percentile method of deviation correction was selected for sampling, the model was selected as 12, the sample size was set to 5,000, and the confidence interval was set to 95%. The moderated mediating effect interval did not contain 0 (LLCI = –1.1301, ULCI = –0.0329, β = –0.5846, and SE = 0.2737), indicating that organic skepticism played a moderated mediating role, thus supporting **H4**. Further, according to the mean and standard deviation, three types of organic skepticism (low, medium, and high) were distinguished. In the interaction of low organic skepticism * health appeal * CA, a mediation effect existed (LLCI = –1.4209, ULCI = –0.2053, β = –0.8336, and SE = 0.3126). In the interaction of high organic skepticism * environmental appeal * CA, a mediating effect existed (LLCI = –1.4209, ULCI = –0.2053, β = –0.7294, and SE = 0.3103).

In sum, the proposed moderated mediating model was supported. CA and benefit appeal directly affect the WTPP for organic food. Simultaneously, it could also predict the willingness to pay through Information persuasiveness, and the mediating role was moderated by organic skepticism.

## Discussion and conclusion

This study conducted three empirical experiments to verify the impact of CA on consumers’ WTPP for organic food. It also examined how benefit types and consumers’ organic skepticism affect CA’s effectiveness.

The findings reveal that, first, matching CA and health appeal increases WTPP, while environmental appeal is not significantly different between comparative and non-comparative advertisements (Study 1). Second, Information persuasiveness plays a mediating role in the interaction between CA and benefit appeal on WTPP (Study 2). Finally, consumers’ organic skepticism moderates the interaction effect between CA and benefit appeal on the persuasiveness of advertising and WTPP (Study 3). For consumers with low organic skepticism, the interaction between CA and health appeal significantly impacts Information persuasiveness. CA with health appeal has the strongest Information persuasiveness. For consumers with high organic skepticism, the interaction between CA and benefit appeal has no significant impact on the persuasiveness of advertising. However, the persuasive power of CA is significantly higher than NCA.

### Implications and contribution

The benefits of organic over ordinary food have not yet been clarified to consumers. Marketers need to develop effective advertising strategies to reduce customer skepticism and stimulate organic consumption (Septianto et al., 2019); CA is effective. Companies often compare organic food with regular food in terms of product details (Zhang et al., 2018). A comparative approach informing consumers of the benefits of organic food may help them decide because them get more useful information (Wang et al., 2021).

This study is the first to explore the role of CA in sustainable product communication, thus broadening the scope of organic advertising research. Meanwhile, the effectiveness of existing CA mainly focuses on product search and experience attributes (Jain et al., 2000). This study focuses on organic food, a credence product, to fill the gap in CA of credence attributes. Research has shown that matching advertising with health claims is more effective for consumers. Health claims may be more related to self-interest; thus, CA is more applicable to interdependent self-construal consumers (Polyorat et al., 2005). Moreover, consumers associate better health with higher prices (Haws et al., 2017). Environmental appeal, regardless of the comparison type, is beneficial to the effectiveness of advertising (Kareklas et al., 2014); thus, organic food brands should use advertising methods that compare ordinary food, such as providing detailed information on how to promote health (e.g., food safety, natural production methods, and nutrition; Molinillo et al., 2020), to enhance their persuasiveness and increase consumers’ WTPP.

The current research chose WTPP rather than the willingness to buy as the dependent variable, essential for organic foods since differences in how organic food is produced require premiums to recoup costs (Berghoef and Dodds, 2013). CA supports the payment of premiums by allowing consumers to clearly understand the comparative advantages of organic food, which is also inspiring for suppliers and advertisers (Molinillo et al., 2020). This will help overcome the inconsistencies in consumers’ behavior regarding sustainable products.

This study contributes to the PKM by revealing that Information persuasiveness plays a mediating role in the interaction between CA and benefit appeal on the WTPP for organic food, and organic skepticism moderates this mediation. CA projecting health claims is more persuasive and is, therefore, more likely to promote consumers’ willingness to pay. Advertising’s credibility and persuasiveness are unavoidable in sustainable product communication (Jäger and Weber, 2020). Highly organic advertising skeptical consumers responded positively to CA. Comparative information containing actual benefits can effectively persuade consumers. The findings provide new insights into PKM, where clearly informing consumers about the health and environmental benefits of organic products can undermine skepticism and increase persuasion. Therefore, organic producers and retailers should use the most authoritative scientific evidence (e.g., Organic food certification system; Wei et al., 2022) and formulate corresponding strategies to promote consumers’ understanding of organic information (such as social media publicity; Liu et al., 2021).

Finally, our study considered consumers’ low/high organic skepticism to explore the boundaries of CA effectiveness. For the high skepticism group, CA can significantly improve Information persuasiveness. This differs from previous studies (Polyorat et al., 2005; Pillai and Goldsmith, 2008), possibly because consumers perceive organic food as being better (Van Doorn and Verhoef, 2015), and CA provides details of the advantages. The interaction between CA and health claims on Information persuasiveness and willingness to pay was stronger for the low skepticism group, demonstrating **H1**’s effectiveness. Generally, CA has a wide range of applications in sustainable product advertising, and its application to health or environmental claims can promote consumer attitudes and healthy consumption. Our study conclusions can help organic restaurants, retailers, and tourist destinations advertise organic food to increase consumers’ willingness to purchase premium prices. For example, encouraging consumers to reduce the consumption of ordinary food to protect the environment, or buying more organic food to promote consumers’ personal health, will help restaurants better promote their products.

### Limitations and further study direction

This study presented some insights for the CA in sustainable product communication, and some limitations can provide directions for future research. First, the brands used herein were fictional, and the findings were more applicable to new brands. Future research could consider existing brands. Second, it is worth discussing whether the object of comparison is the same category or not, because in reality there are a lot of advertisements comparing organic food with products that consumers are familiar with. Third, this study only discusses limited mediating variables and moderating factors, but there may be other variables that can be used to explain the effect of CA, such as consumers’ food safety concerns, competitive orientation, and comparative advertising strategies; Finally, this study used the Chinese context; future cross-cultural research is encouraged.

## Data availability statement

The raw data supporting the conclusions of this article will be made available by the authors, without undue reservation. Further inquiries can be directed to the corresponding author.

## Author contributions

WY: conceptualization, project administration, supervision, and funding acquisition. XH: conceptualization, formal analysis, investigation, data curation, writing—original draft, writing—review and editing, and visualization. FC: data curation, investigation, writing—original draft, and writing—review and editing. All authors contributed to the article and approved the submitted version.

## Footnotes

1 The National People’s Congress of the People’s Republic of China (2018, November 5). *Advertising Law of the People’s Republic of China.* http://www.npc.gov.cn/npc/c12435/201811/c10c8b8f625c4a6ea2739e3f20191e32.shtml

2
www.sojump.com
